# Preliminary outcomes of DEB-TACE loaded with raltitrexed in the treatment of unresectable or recurrent hepatocellular carcinoma

**DOI:** 10.1186/s40644-023-00534-1

**Published:** 2023-02-22

**Authors:** Yonghua Bi, Yang Wang, Wenguang Zhang, Huibin Lu, Jianzhuang Ren, Xinwei Han

**Affiliations:** Department of Interventional Radiology, the First Affiliated Hospital of Zhengzhou University, No.1, East Jian She Road, Zhengzhou, 450052 China

**Keywords:** Carcinoma, Hepatocellular, Chemoembolization, Therapeutic, Microspheres, Raltitrexed

## Abstract

**Purpose:**

Raltitrexed shows therapeutic effects and safety in many types of malignant tumors. However, reports of the clinical outcomes of raltitrexed-based transarterial chemoembolization (TACE) or drug-eluting beads TACE (DEB-TACE) in the treatment of hepatocellular carcinoma (HCC) are rare. We aim to report the preliminary outcomes of DEB-TACE loaded with raltitrexed in patients with unresectable or recurrent HCC.

**Methods:**

From June 2018 to March 2020, 29 patients with unresectable or recurrent HCC were recruited from our department and treated by DEB-TACE loaded with raltitrexed. Overall survival and progression-free survival were the primary end points. Tumor response was investigated by using the modified response evaluation criteria in solid tumors (mRECIST) criteria.

**Results:**

A total of 49 sessions of DEB-TACE were performed, with a technique success rate of 100%. The overall response rate and disease control rate at 1, 3, and 6 months after DEB-TACE were 72.0% and 96.0%, 57.1% and 85.7%, 47.6% and 66.7% respectively. The median progression-free survival and overall survival was 25.7 and 33.9 months, respectively. The 6-, 24- and 36-month overall survival rates were 88.4%, 66.3% and 46.3%, respectively. Minor complications were observed in 17 patients (58.6%), with no treatment-related mortality or severe adverse events. The most common treatment-related complications were abdominal pain (41.4%) and elevated ALT/AST (27.6%).

**Conclusion:**

DEB-TACE loaded with raltitrexed is suggested as a safe, feasible, efficacious palliative regimen in unresectable or recurrent HCC patients.

## Introduction

Hepatocellular carcinoma (HCC) is one of the most common leading causes of cancer-related mortality in the world [1, 2]. Only about one-fifth of patients are diagnosed with early-stage HCC and can be treated with curative treatments, while most patients are late-stage HCC and its prognosis remains poor. Transarterial chemoembolization (TACE) has been considered as an efficacious palliative treatment to improve survival for unresectable HCC patients [3, 4]. Conventional TACE (cTACE) and drug-eluting bead TACE (DEB-TACE) are two principal TACE techniques [5]. In cTACE, a drug is carried by lipiodol and then is administered as an arterial embolization. There is still no standardization of the embolization endpoint or the choice of the drug. A number of adverse effects are observed with the most common drugs used during cTACE, such as doxorubicin, fluorouracil, and oxaliplatin, indicating that alternative agents are needed [6]. Raltitrexed, a quinazoline antifolate thymidylate synthase inhibitor [7], shows therapeutic effects and safety in a number of malignant tumors including breast and colorectal cancers [8–10], as well as HCC [11, 12].

As a novel drug-delivering device, DEB-TACE is characterized by arterial embolization of microspheres loaded with drug. Technique standardization is ensured by calibrating the same amount of drug loaded into the microspheres, and thus shows an advantage of increased local chemotherapy concentration and sustained release of anti-tumor drugs [13, 14]. Although cTACE and DEB-TACE show equal safety and effectiveness, DEB-TACE has the advantage of causing less abdominal pain after the procedure [15]. Currently, reports of the clinical outcomes of DEB-TACE loaded with raltitrexed in the treatment of HCC are quite rare. Yang et al.[16] investigated the role of raltitrexed in combination with oxaliplatin/epirubicin, rather than as a single agent, making it difficult to distinguish its efficacy from other drugs. In this study, we aim to report the preliminary outcomes of DEB-TACE loaded with raltitrexed in patients with unresectable or recurrent HCC.

## Patients and methods

### Selection of patients

**Fig. 1:**
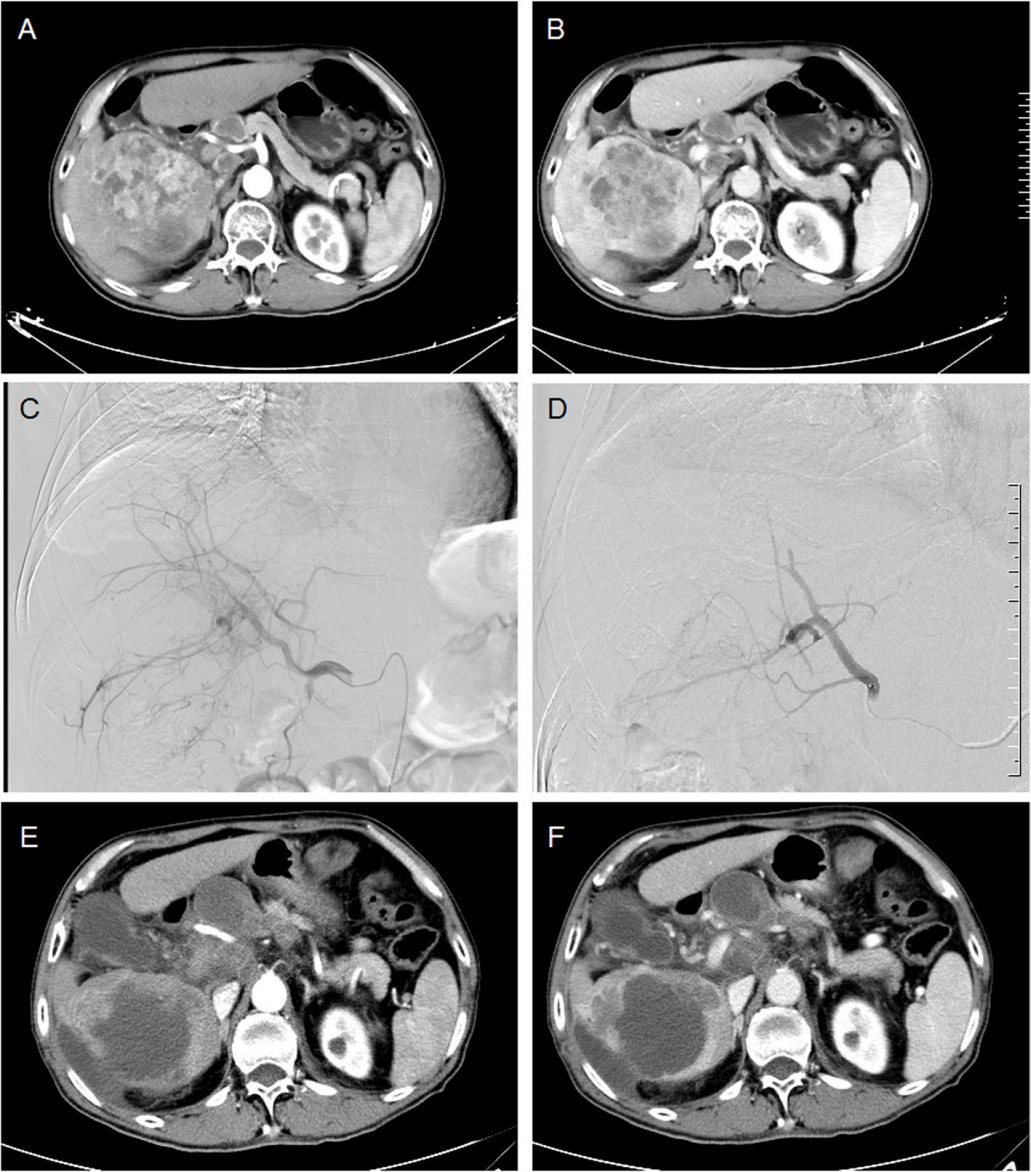
A 68-year male treated by DEB-TACE for advanced primary HCC. **A**-**B** CT examination on admission revealed HCC and portal venous tumor emboli. **C** A huge tumor staining was shown and the right hepatic artery was its blood supply artery. **D** The right hepatic artery was embolized by 300-500 μm raltitrexed-loaded beads. **E**–**F** The tumor was found to shrink after 4 months' follow-up, but died of tumor progression 38.3 months later

This retrospective study was conducted in accordance with the Declaration of Helsinki and was approved by the Institutional Review Board and the Human Ethics Committee of our hospital. Written informed consent for the DEB-TACE protocol was obtained from all enrolled patients. Patients were recruited from our department between June 2018 and March 2020 and were diagnosed primary HCC, no matter unresectable or recurrent tumor. Contrast-enhanced computed tomography (CT) and/or magnetic resonance imaging (MRI) were carried out at baseline according to the international standards [17]. The typical imaging features to achieve the diagnosis of HCC includes arterial phase hyperenhancement and washout on portal venous and/or delayed phases in the setting of a cirrhotic liver (Figs. 1AB, 2AB, 3A and 4A). The inclusion criteria: > 18 years; a life expectancy > 3 months; Barcelona Clinic Liver Cancer (BCLC) stage B or C; Child–Pugh class A or B. Exclusion criteria: allergy to study drugs; liver metastasis cancer; severe cardiovascular comorbidities including unstable angina, myocardial infarction or uncontrolled hypertension; coagulopathy or bleeding diathesis; BCLC stage D; Child–Pugh class C; Tumor occupancy > 70%.

**Fig. 2:**
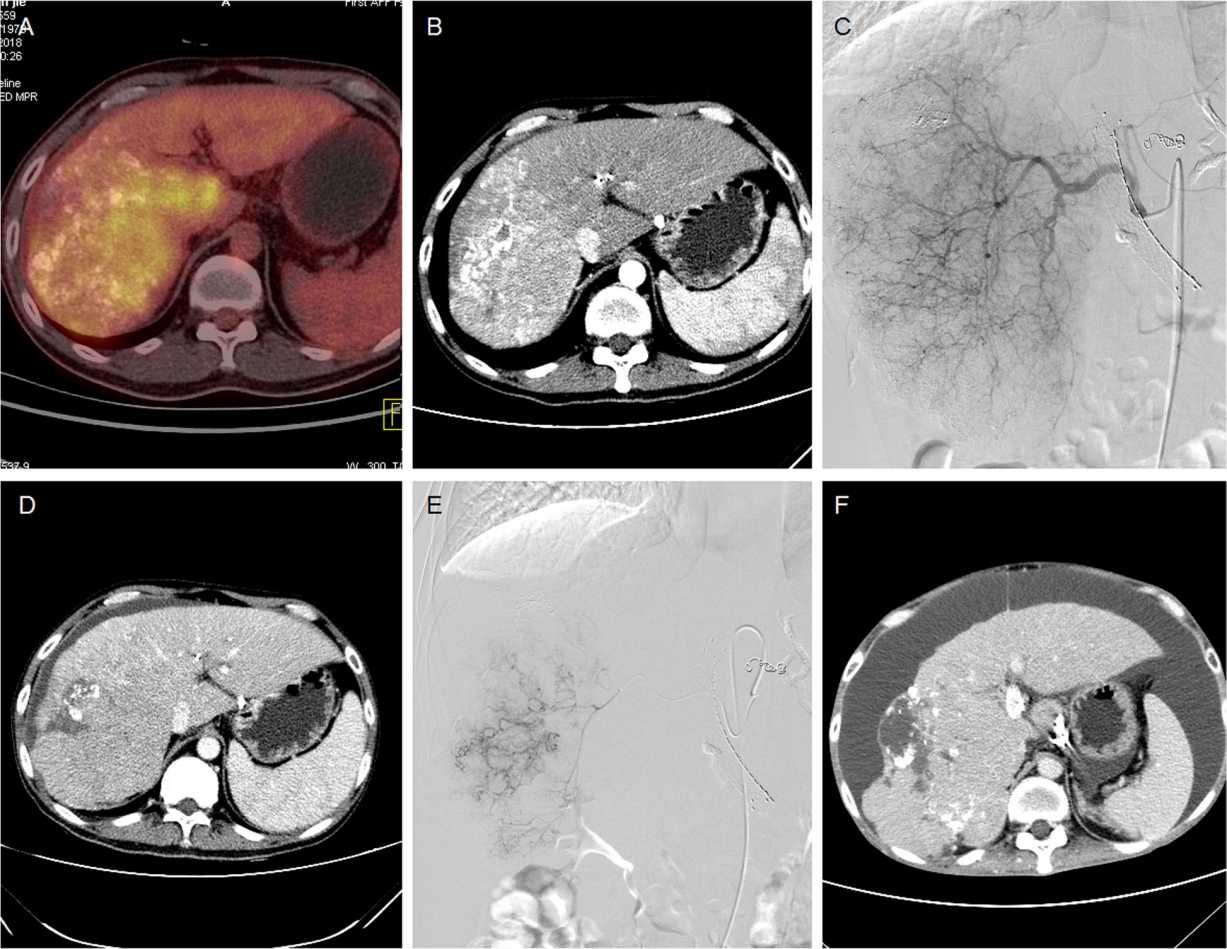
A 41-year male treated by DEB-TACE for advanced primary HCC. **A**-**B** CT revealed a large tumor of 133.2 mm in right liver one month after conventional TACE. **C** The tumor artery was superselectively incubated and embolized by 300-500 μm raltitrexed-loaded beads. **D** CT revealed a shrunk tumor 1.3 months after first DEB-TACE. **E** A second DEB-TACE was performed with 300-500 μm raltitrexed-loaded beads. **F** The tumor was found to shrink after 5.1 months' follow-up, but died of disease progression 7.5 months later

**Fig. 3:**
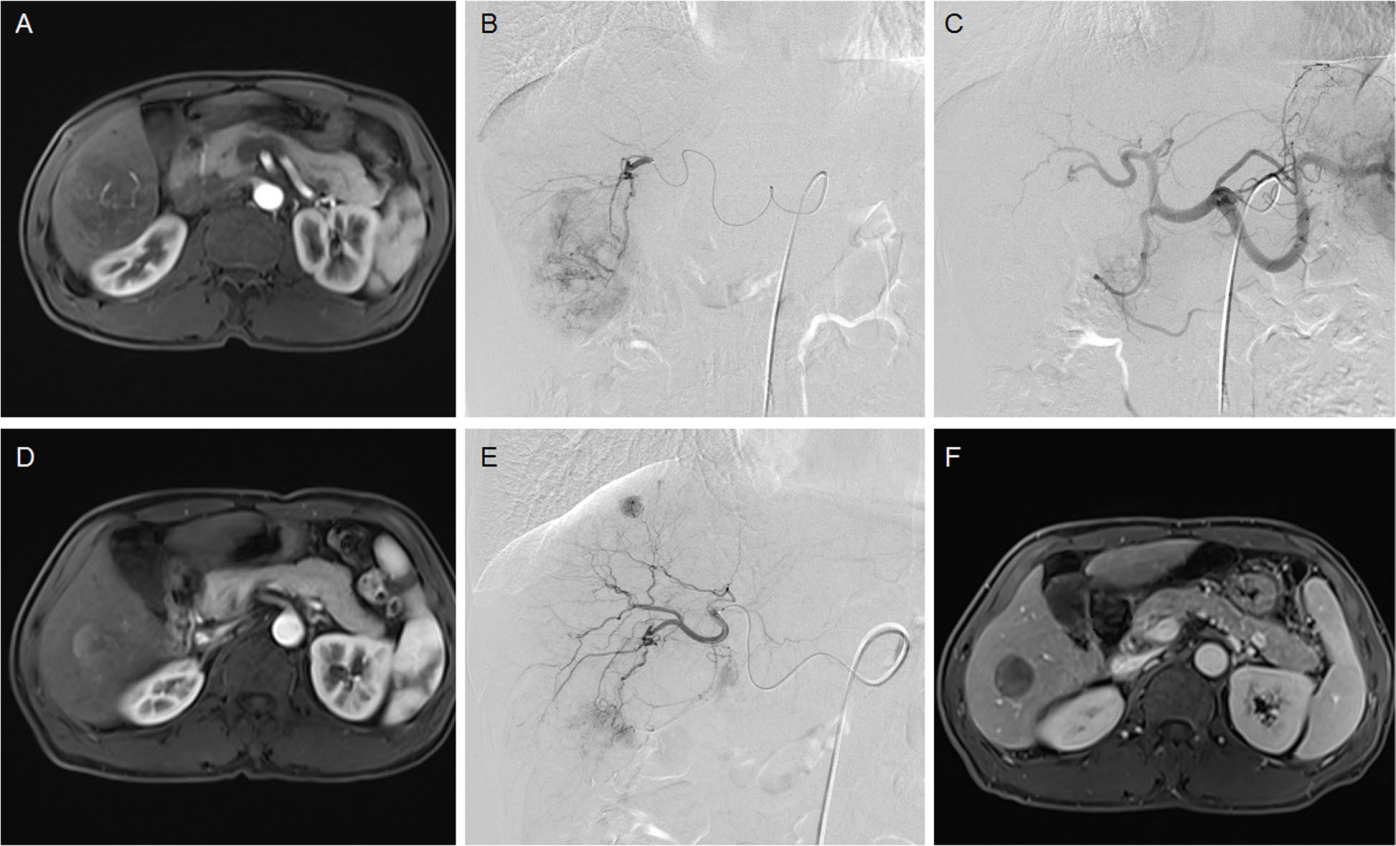
A 53-year male treated by DEB-TACE for advanced primary HCC. **A** MRI preoperative examination revealed a large tumor of 61.2*48.5 mm in right liver. **B**-**C** The tumor artery was superselectively incubated and embolized by 300-500 μm raltitrexed-loaded beads. **D** MRI revealed a shrunk tumor 1.2 months later. **E** A small tumor was found and embolized. **F** The tumor was found to shrink after 8.0 months' follow-up, and still alive after 34.0 months

**Fig. 4:**
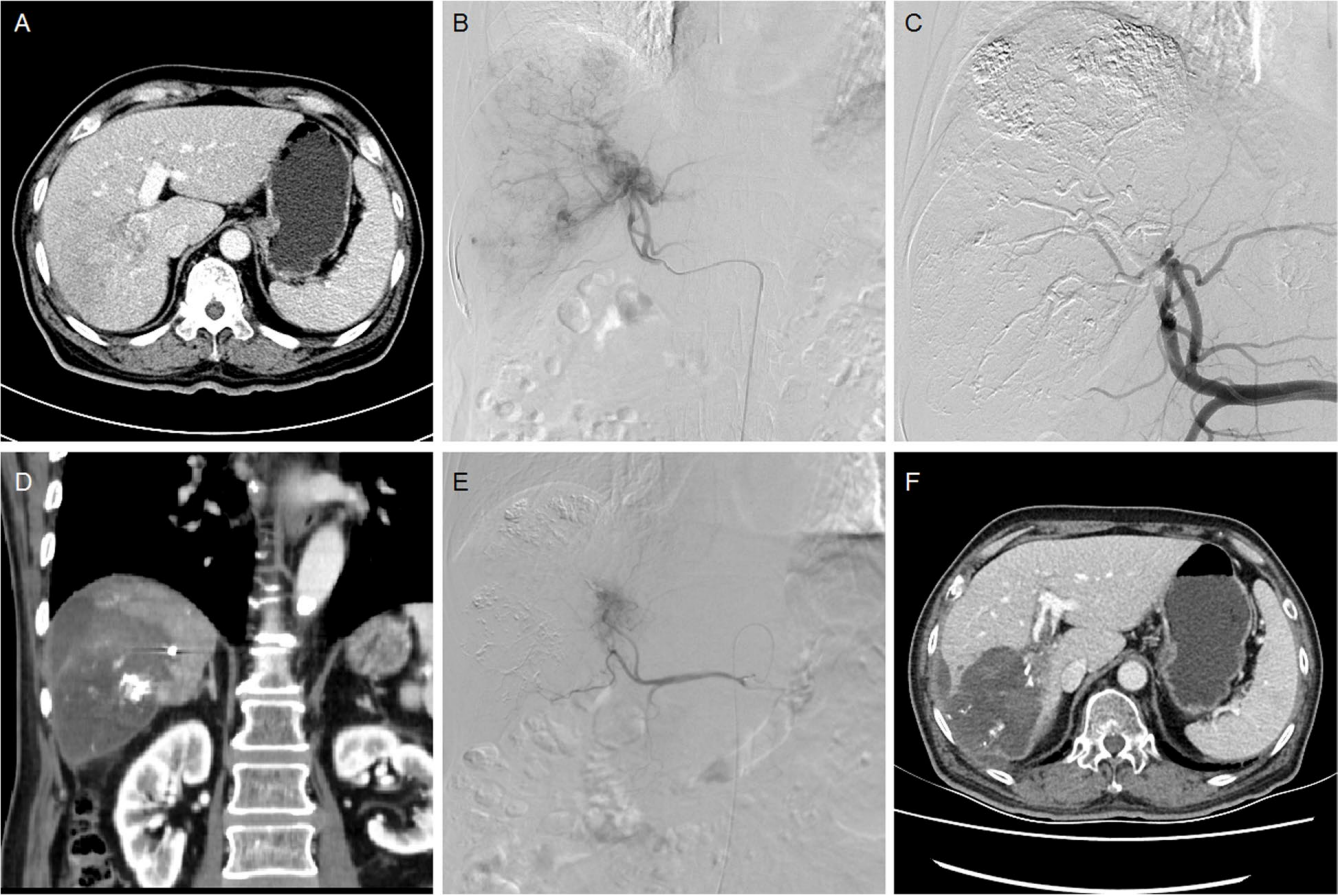
A 65-year male treated by DEB-TACE for advanced primary HCC. **A** CT revealed HCC and portal venous tumor emboli. **B** A huge tumor staining was shown and the right hepatic artery was its blood supply artery. **C** The right hepatic artery was embolized by 100–300 μm raltitrexed-loaded beads. **D** CT revealed a shrunk tumor 3.5 months later. **E** A small tumor was found and embolized. **F** The tumor was found to shrink after 5.0 months' follow-up, and still alive after 34.7 months

### DEB-TACE procedures

The femoral artery was accessed after local anesthesia and a 5F-Cobra catheter (Terumo, Japan) was introduced. Tumor-feeding vessels were catheterized and a 2.7-F microcatheter (Progreat, Terumo, Japan) was used for super-selection. The dose of raltitrexed is calculated based on the Body Mass Index, and is generally 4 mg. Raltitrexed (4 mg) was pre-loaded into microspheres (CalliSpheres, Jiangsu Hengrui Medicine Co. Ltd., Jiangsu, China) for 30 min, and then mixed with iodixanol (ratio 1:1). A bottle of 100–300 μm or 300–500 μm raltitrexed-loaded microspheres was slowly injected into the tumor-feeding vessels. Polyvinyl alcohol particles (Merit, American) or gelatin sponge particles were used if one bottle of microspheres embolization was insufficient (Figs. 1CD, 2CE, 3BCE and 4BCE).

### Efficacy evaluation

The primary end points were overall survival and progression-free survival. Tumor responses were assessed using the modified response evaluation criteria in solid tumors (mRECIST) [18]. Patients were subjected to a follow-up and a CT/MRI scan about 1, 3 and 6 months after DEB-TACE, and then every 2 to 3 months thereafter (Figs. 1EF, 2DF, 3DF and 4DF). The next session treatment was recommended for all patients until the patient achieved a complete response or death. All CT and MRI data were evaluated by two experienced radiologists to minimize variability [19]. Patients were followed up by phone calls, and the last follow-up date was 10 January 2022. One patient was lost to follow-up.

### Safety evaluation

The preoperative laboratory tests include routine hematologic tests, liver function, renal function, blood coagulation function, and electrolytes. These tests were repeated about 5 to 7 days after the DEB-TACE procedure to assess toxicity. Safety and toxicity, assessed for adverse events according to the National Cancer Institute Common Toxicity Criteria (version 3.0) [20], were the secondary end points.

## Results

### Patient characteristics

We enrolled 29 patients in this study, including 25 males and 4 females (mean age 59.2 ± 11.3 years, range 37–84 years). Detailed baseline demographics and disease characteristics are shown in Table 1. Four patients (13.8%) had recurrent HCC and 25 patients (86.2%) had unresectable HCC. A single tumor was found in 15 (51.7%) patients, and 4 (13.8%) patients had two nodules, and the remaining 10 (34.5%) patients had at least of 3 nodules. Intrahepatic and extrahepatic metastases were present in 8 patients (27.6%) and 9 patients (31.0%), respectively. A tumor in portal vein was found in 9 patients (31.0%). Fifteen patients complained of abdominal swelling or pain, and the median duration of symptom was 1.5 (interquartile range [IQR] 0.7, 17.0) months. All 7 Child Purge B were included for DEB-TACE, including 3 patients with Child-Turcotte-Pugh (CTP) score of 7, 2 patients with a CTP score of 8 and 2 patients with CTP score of 9.

**Table 1:** Patient characteristics at admission

Parameters	Data
Male, n (%)	25 (86.2%)
Mean age, years	59.2 ± 11.3
Lesion types	
Right liver	16 (55.2%)
Left liver	2 (6.9%)
Whole liver	11 (37.9%)
Median duration of symptom, months	1.0 (0.6, 6.0)
Recurrent/unresectable HCC	4 (13.8%)/25 (86.2%)
Systemic chemotherapy	1 (3.4%)
Targeted therapy	13 (44.8%)
Etiology HBV/HCV	25 (86.2%)/2 (6.9%)
BCLC stage B/C	7 (24.1%)/22 (75.9%)
Child–Pugh class A/B	22 (75.9%)/7 (24.1%)
One/two/multiple tumors	15 (51.7%)/4 (13.8%)/10 (34.5%)
Portal venous tumor emboli	9 (31.0%)
Local or distant metastasis	14 (48.3%)/3 (10.3%)
Tumor diameter, mm	91.6 ± 41.2

*HBV* Hepatitis B Virus, *HCV* Hepatitis C Virus, *BCLC* Barcelona Clinic Liver Cancer

### DEB-TACE Treatments

A total of 49 sessions of DEB-TACE were administered to 29 patients, and the mean of DEB-TACE cycle was 1.7 ± 1.2. Eighteen patients completed one cycle of DEB-TACE, and the remained 11 patients completed at least two cycles of DEB-TACE. The median interval between the first and second session was 1.7 (IQR 1.4, 3.1) months. All DEB-TACE procedures were successfully performed, with a technique success rate of 100%. Except for 6 sessions of DEB-TACE with microspheres of 100–300 μm, the remaining 43 sessions of DEB-TACE used microspheres of 300–500 μm in diameter. All patients received a bottle of beads, and additional embolization was performed by polyvinyl alcohol of 350–560 μm during 6 sessions, gelatin sponge particles of 350–560 μm during 11 (22.4%) sessions, or embolization microsphere of 300–500 μm during 2 sessions of DEB-TACE. The mean cost of hospitalization was 5.3 × 10^4^￥ and median total cost was 7.1 (range 5.3–10.8) × 10^4^￥.

### Other related treatments

Nineteen patients (65.5%) received other related treatments. Nine patients underwent cTACE, which was done before DEB-TACE in 6 patients and after DEB-TACE for next session in only 3 patients (Table 2). Nine patients received thermal ablation, which was done in one patient for bone metastases. Seven other patients had received previous thermal ablation before the DEB-TACE procedure. Only one patient received thermal ablation for a residual partial lesion after DEB-TACE due to the lack of blood supply. One patient had a main portal vein occlusion and portal hypertension due to tumor thrombi formation. After portal stent placement (12 mm × 100 mm, Bard, Tempe, AZ, USA) and brachytherapy with Iodine-125 seeds strands (20 seeds), portal blood flow was successfully restored to reduce portal pressure. In addition to this patient with portal vein particle insertion, three other patients received Iodine-125 seeds placement, one for bone metastases, one for retroperitoneal metastatic lymph nodes, and in one placement in liver lesion was done prior to the DEB-TACE treatment.

**Table 2:** Clinical data on DEB-TACE

Variables	Data
Microsphere of 100–300/300-500 μm	6 (12.2%)/43 (87.8%)
Polyvinyl alcohol particles	6 (12.2%)
Gelatin sponge particles	11 (22.4%)
Embolization microspheres	2 (4.1%)
Median inpatient duration, months	10.0 (7.0, 13.0)
Total cost of hospitalization, × 10^4^￥	7.1 (5.3, 10.8)
Mean session of DEB-TACE	1.7 ± 1.2
Complications, n (%)	17 (58.6%)
Fever	4 (13.8%)
Nausea and/or vomiting	5 (17.2%)
Abdominal pain	12 (41.4%)
Raised ALT/AST	8 (27.6%)
Hematoma	1 (3.4%)
Other related treatments, n (%)	19 (65.5%)
cTACE	9 (31.0%)
Portal vein stenting	1 (3.4%)
^125^I seeds implantation	4 (13.8%)
Thermal ablation	9 (31.0%)

*ALT* Alanine aminotransferase, *AST* Aspertate aminotransferase

### Tumor response

Tumor enhancement decreased significantly after DEB-TACE. The overall response rates at 1, 3, and 6 months were 72.0%, 57.1%, 47.6% respectively. The disease control rates at 1, 3, and 6 months were 96.0%, 85.7% and 66.7% respectively (Table 3).

**Table 3:** Local tumor response 1, 3 and 6 months after DEB-TACE procedure

Response	1 month	3 months	6 months
Complete response	7 (28.0%)	6 (28.6%)	6 (28.6%)
Partial response	11 (44.0%)	6 (28.6%)	4 (19.0%)
Stable disease	6 (24.0%)	6 (28.6%)	4 (19.0%)
Progressive disease	1 (4.0%)	3 (14.3%)	7 (33.3%)
Overall response rate	18 (72.0%)	12 (57.1%)	10 (47.6%)
Disease control rate	24 (96.0%)	18 (85.7%)	14 (66.7%)

### Survival

The mean follow-up duration was 23.2 ± 18.4 months. The median progression-free survival was 25.7 months, and the 6-, 24- and 36-month progression-free survival rates (PFS) were 72.3%, 51.9% and 37.5%, respectively. The median overall survival was 33.9 months, and the 6-, 24- and 36-month overall survival rates were 88.4%, 66.3% and 46.3%, respectively. A total of 14 patients (48.3%) received other concomitant treatments. Specifically, one patient received systemic chemotherapy and 13 patients received targeted therapy with solafini (0.4 g BID, po., *n* = 4) or apatinib (0.25–0.75 g QD, po., *n* = 9). However, there was no significant difference in overall survival or PFS between the two groups with or without targeted therapy. This may be related to the small sample size, and the oral administration of two different targeted drugs.

### Safety and toxicity

Minor complications were observed in 17 patients (58.6%), with no treatment-related mortality or severe adverse events. The most common treatment-related nonhematologic complications were abdominal pain (41.4%) and elevated ALT/AST (27.6%). All reported toxicities were grades 1 and 2.

## Discussion

In southeast Asia, HCC shows the highest incidence and hepatitis B virus transmission is considered the major risk factor [21]. About 80% of HCC patients are diagnosed in the intermediate or advanced stage. The introduction of hepatospecific contrast media for MRI and new diagnostic algorithms for HCC will allow identification of smaller lesions and therefore also in earlier stages in the near future [22]. However, TACE is recommended as the first-line therapy for Barcelona Clinic Liver Cancer (BCLC) tumor stage B patients [23], and only systemic therapy is considered to be recommended for advanced BCLC C HCC in Western countries, while China and other Asia countries extends the indication for advanced BCLC C HCC [24], and still recommend TACE for patients with portal vein invasion, even at the main trunk [25].

As one of the most widely used chemotherapeutic agents, doxorubicin is considered a broad-spectrum, highly powerful for HCC treatment. Unfortunately, its application is compromised by dose-dependent cardiotoxicity [26]. Doxorubicin-loaded nanoparticles may be used as a possible drug delivery system for reducing this side effects [26]. Besides, monoclonal antibodies against tyrosine kinase receptors, antiangiogenic drugs, and tyrosine kinase inhibitors also elicit cardiotoxic effects, such as sunitinib and trastuzumab [27].

Currently, oxaliplatin in combination with doxorubicin or fluorouracil is usually administered during the TACE procedure. However, these drugs are not suitable for patients with previous cardiotoxicity or high cardiologic risk factors [28], indicating the need for a new kind of agent. Raltitrexed, a thymidylate synthase inhibitor [9], is effective in many types of tumor types, with or without oxaliplatin or doxorubicin [8]. Raltitrexed may theoretically serve as a substitute for fluorouracil due to the lack of cardiac toxicity [6, 28]. Currently, very few studies have reported the efficacy and safety of raltitrexed-based TACE or DEB-TACE in the treatment of advanced HCC.

It has been reported that raltitrexed-based cTACE show a superior objective response rate than that of doxorubicin and fluorouracil-based cTACE in patients with unresectable HCC. Zhao et al. [12] reported that raltitrexed plus oxaliplatin-based cTACE is a safe and efficacious regimen in unresectable HCC, and the median overall survival and median PFS were 13.4 and 6.7 months, respectively. He J et al.[29] reported that raltitrexed‐based cTACE is effective and safe for intermediate and advanced HCC using real-world evidence. The median overall survival was 10.0 months and the 6-month and 2-year overall survival rates were 78.2% and 17.4%, respectively. In our study, the median overall survival was 33.9 months and the 6-month and 2-year overall survival rates were 88.4% and 66.3%, respectively. Our data were better than those of the aforementioned studies, indicating that DEB-TACE may have a better efficacy than cTACE.

As a novel drug-delivering device, DEB-TACE showed the advantage of an increased local chemotherapy concentration and sustained release of anti-tumor drugs [30, 31]. Microspheres loaded with pirarubicin was often used for the treatment of HCC. When compared to Microspheres beads loaded with pirarubicin (60 mg or 80 mg)[32], our results showed a similar PFS (25.7 vs. 25.3 months) but longer overall survival (33.9 vs. 27.8 months). Besides, our data showed improved outcomes than DEB-TACE loaded with oxaliplatin for unresectable or recurrent HCC, in which, the median overall survival was 18.8 months and PFS was 5.9 months [33]. Yang et al. [16] reported that DEB-TACE loaded with epirubicin and raltitrexed improves the clinical outcomes and survival rate in patients with intermediate and advanced HCC. At 6 months after the DEB-TACE, the overall response rate (82%) was also similar to our results.

In this study, we also wanted to evaluate whether DEB-TACE loaded with raltitrexed was tolerable and safe in patients with unresectable or recurrent HCC. Our data showed that the most common treatment-related complication was abdominal pain (41.4%), similar to a previous study [11]. There was no treatment-related mortality or severe adverse events. All reported toxicities were grades 1 and 2, which were manageable and reversible. In addition, 27.6% of patients had elevated ALT/AST after the procedure. Granito et al. [34] reported that post-treatment transient hypertransaminasemia can be an accurate predictor of a complete response to superselective cTACE, representing a simple tool to guide treatment the strategy of HCC patients. However, in our study, patients that presented an elevated ALT/AST did not show significantly different overall response rates at 6 months when compared to those without elevated ALT/AST (*p* = 0.36).

Moreover, DEB-TACE has higher immediate costs than cTACE. Cucchetti et al. reported that total costs were €11,418 and €10,389 for DEB-TACE and cTACE, respectively [35]. Many interventional radiology centers worldwide continue to use cTACE because of the cost savings [5]. However, the direct incremental costs of DEB-TACE can be acceptable with respect to cTACE, relying on financial resources available from the payer perspective, mainly depending on the better quality of life and shorter in-hospital stay [35]. Our study showed a similar total cost as the report of Cucchetti A et al. [35].

Our study has some limitations. This was a retrospective study with a limited sample size and was conducted in a single center. Both recurrent and unresectable HCC patients were enrolled, which is not conducive to drawing a clear conclusion. Seven patients with Child Purge B were included for DEB-TACE, they may have more chances of decompensation in theory. The limited sample size of recurrent HCC does not allow for a statistically powered subgroup analysis.

## Conclusion

Our preliminary results showed that DEB-TACE loaded with raltitrexed is safe and tolerable in patients with unresectable HCC; further clinical studies are warranted.

## Data Availability

All the data concerning this study are available from the corresponding author.

## Abbreviations

TACE: Transarterial chemoembolization
DEB-TACE: Drug-eluting beads transarterial chemoembolization
RECIST: Response evaluation criteria in solid tumors
HCC: Hepatocellular carcinoma
CT: Computed tomography
MRI: Magnetic resonance imaging
ALT: Alanine aminotransferase
AST: Aspertate aminotransferase
CTP: Child-Turcotte-Pugh
IQR: Interquartile range
